# The first complete genome of the simian malaria parasite *Plasmodium brasilianum*

**DOI:** 10.1038/s41598-022-20706-6

**Published:** 2022-11-17

**Authors:** Marko Bajic, Shashidhar Ravishankar, Mili Sheth, Lori A. Rowe, M. Andreina Pacheco, Dhruviben S. Patel, Dhwani Batra, Vladimir Loparev, Christian Olsen, Ananias A. Escalante, Fredrik Vannberg, Venkatachalam Udhayakumar, John W. Barnwell, Eldin Talundzic

**Affiliations:** 1 Association of Public Health Laboratories, Silver Spring, MD USA; 2 Malaria Branch, Division of Parasitic Diseases and Malaria, Center for Global Health, Centers for Disease Control and Prevention, Atlanta, GA USA; 3 Fred Hutchinson Cancer Research Center, Seattle, WA USA; 4 Biotechnology Core Facility Branch, Division of Scientific Resources, Centers for Disease Control and Prevention, Atlanta, GA USA; 5 Virus Characterization Isolation Production and Sequencing Core, Tulane National Primate Research Center, Covington, LA USA; 6 Biology Department/Institute of Genomics and Evolutionary Medicine (iGEM), Temple University, Philadelphia, PA USA; 7 Center for Integrative Genomics at Georgia Tech, Georgia Institute of Technology, Atlanta, GA USA

**Keywords:** Comparative genomics, Genome evolution, Malaria

## Abstract

Naturally occurring human infections by zoonotic *Plasmodium* species have been documented for *P. knowlesi*, *P. cynomolgi*, *P. simium*, *P. simiovale*, *P. inui*, *P. inui*-like, *P. coatneyi*, and *P. brasilianum*. Accurate detection of each species is complicated by their morphological similarities with other *Plasmodium* species. PCR-based assays offer a solution but require prior knowledge of adequate genomic targets that can distinguish the species. While whole genomes have been published for *P. knowlesi*, *P. cynomolgi*, *P. simium*, and *P. inui*, no complete genome for *P. brasilianum* has been available. Previously, we reported a draft genome for *P. brasilianum*, and here we report the completed genome for *P. brasilianum*. The genome is 31.4 Mb in size and comprises 14 chromosomes, the mitochondrial genome, the apicoplast genome, and 29 unplaced contigs. The chromosomes consist of 98.4% nucleotide sites that are identical to the *P. malariae* genome, the closest evolutionarily related species hypothesized to be the same species as *P. brasilianum*, with 41,125 non-synonymous SNPs (0.0722% of genome) identified between the two genomes. Furthermore, *P. brasilianum* had 4864 (82.1%) genes that share 80% or higher sequence similarity with 4970 (75.5%) *P. malariae* genes. This was demonstrated by the nearly identical genomic organization and multiple sequence alignments for the merozoite surface proteins *msp3* and *msp7*. We observed a distinction in the repeat lengths of the circumsporozoite protein (CSP) gene sequences between *P. brasilianum and P. malariae*. Our results demonstrate a 97.3% pairwise identity between the *P. brasilianum* and the *P. malariae* genomes. These findings highlight the phylogenetic proximity of these two species, suggesting that *P. malariae* and *P. brasilianum* are strains of the same species, but this could not be fully evaluated with only a single genomic sequence for each species.

## Introduction

Human malaria, caused by protozoan parasites of the *Plasmodium* genus*,* is prevalent across many regions of the world, with the highest burden in Africa, followed by Asia, and then the Americas^1^. There are over 100 species of *Plasmodium*, but only *Plasmodium falciparum*, *P. vivax*, *P. ovale curtisi*, *P. ovale wallikeri*, and *P. malariae* are considered human malaria parasites. There are non-human malaria parasites that cause zoonotic infections in humans, among them *P. knowlesi* is known to infect humans residing in Southeast Asia^2,3^. Recent reports have identified several other zoonotic infections in humans^4^. Human malaria caused by *P. brasilianum* has also been reported from the Amazon basin region of South America^5^.

**Figure 1:**
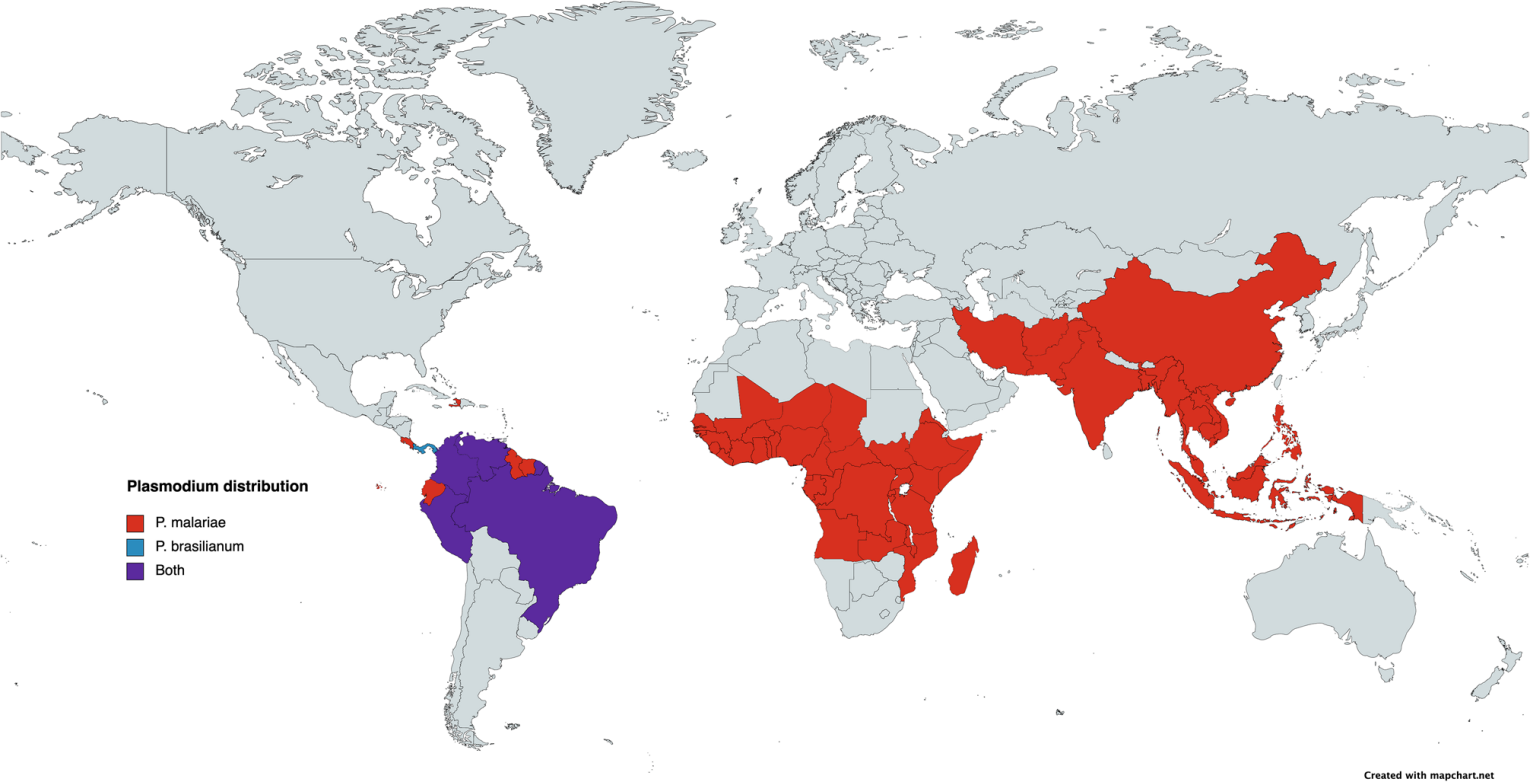
Global distribution of *P. malariae* and *P. brasilianum*. Reported distributions of *P. malariae* (red), *P. brasilianum* (blue), or both (purple) globally based on previous publications ^6,7^. While *P. brasilianum* has been reported in Central America in the past, there have been no recent reports of its presence or risk to humans outside of South America.

*Plasmodium brasilianum* is a malaria parasite of non-human primates found thus far in 13 genera and 36 species of New World monkeys across Central and South America (Fig. 1)^6,7^. Previous studies have shown strong similarities in the morphology and 18 s rRNA, mtDNA, and several surface protein gene sequences between *P. brasilianum* and *P. malariae*^8,9^. Furthermore, the amino acid sequence composition and protein structure are similar in the circumsporozoite protein (CSP) and merozoite surface protein 1 (MSP1) between the two species^5,10^.

These similarities have led to the suggestion that the two species are indeed the same species, with *P. brasilianum* originating from a spillover of *P. malariae* into nonhuman primates^5^. However, this hypothesis could not be thoroughly evaluated due to the lack of well-curated reference genomes for either species. The first complete genome for *P. malariae* highlighted the similarity between the two species^7^. To date, there has not been a whole reference genome available for *P. brasilianum.* A draft genome for *P. brasilianum* generated previously by our team from short-read sequencing revealed that the mitochondrial and apicoplast genomes of the two species were 99.7% and 99.6% identical between *P. brasilianum* and *P. malariae*, respectively^11^.

In this paper, we extend our previous effort using a combination of optical mapping, longer read PacBio sequencing, and Illumina short-read sequencing to generate the first complete reference genome of *P. brasilianum*. Using the previously published reference genome for the human infecting parasite *P. malariae*^7^, we compared the two genomes, which reveal strong genetic similarities. This is readily apparent in our comparisons of the *msp3* and *msp7* regions between the two species. The multigene members of each gene family, comprising 12 and 7 members, respectively, were organized in the same orientation in both genomes, with several nucleotide changes comprising the major differences observed.

Overall, we were not able to determine whether *P. brasilianum* and *P. malariae* are the same species from only a single genome from each species, but the availability of the *P. brasilianum* reference genome will facilitate the identification of species-specific diagnostic targets for detecting zoonotic transmission and will help drive future research related to *Plasmodium* evolution.

## Results

### Genome assembly and annotation

The host depleted QC passed reads from the two Illumina MiSeq libraries, and the PacBio RSII long reads were assembled using the hybrid assembly approach implemented in MaSuRCA^12^. The initial assembly consisted of 148 contigs. Circlator^13^ and IPA (https://github.com/ThomasDOtto/IPA) were used to merge overlapping contigs and generate scaffolds. ABACAS^14^ was used to port over chromosome names from equivalent contigs annotated in the *P. malariae* genome^7^. The assembled scaffolds were polished and SNPs and InDels with respect to *P. malariae* were identified using PILON^15^. The final assembly contained the 14 chromosomes, the mitochondrial genome, the apicoplast genome, and 29 unplaced contigs (Fig S1). To date, this is the most complete genome that has been generated for *P. brasilianum*. The assemblies of the 14 chromosomes were further optimized using optical mapping data; we observed near telomere-to-telomere assembly for 9 of 14 chromosomes when comparing the optical maps at the AfIII break-sites. We report the longest contiguous sequence for the remaining five chromosomes (PB01, PB02, PB03, PB04, PB06). The reported chromosomes show a high degree of similarity when compared with the *P. malariae* chromosomes. Pairwise sequence alignments between the two genomes show no structural differences (data not shown).

### Genomic characteristics of *Plasmodium brasilianum*

The final assembled *P. brasilianum* genome is 31.4 Mb in length, consisting of 14 chromosomes, the mitochondrial genome, the apicoplast genome, and 29 unplaced contigs (Table 1). The contiguous genomes are 29.1 Mb in size and have 24.7% GC content, whereas the unplaced contigs all together are 2.29 Mb in size and have 20.6% GC content (Table S1). In contrast, the *P. malariae* genome^7^ is 33.6 Mb in length and has 47 unplaced contigs. The *P. malariae* contiguous genomes are 29.6 Mb in size and have 24.9% GC content, whereas the unplaced contigs all together are 4.04 Mb in size and have 18.9% GC content. If only the 14 mapped chromosomes and organellar genomes are considered, the two genomes vary in size by 0.461 Mb (1.57%) and GC content by 0.11%, making them 98.4% similar in size and 99.9% similar in GC content (Table S1).

**Table 1:** Assembly and annotation summary for *P. brasilianum* and *P. malariae.*

Feature	*P. brasilianum*	*P. malariae*
Genome size (Mb)	31.4	33.6
Contigs	14 (29)	14 (47)
GC content (%)	24.8	24.4
Genes (n)	5923	5942
Pseudogenes (n)	N/A	637
*pir*	144	137
*stp1*	100	109
*tryptophan-rich*	31	28
*etramp*	9	7
*phist*	20	29
*fam-l*	213	289
*fam-m*	157	194

The genome size, number of chromosomes (scaffolds), GC content, and number of genes and pseudogenes is listed for *P. brasilianum* and *P. malariae*. The final 7 rows list the number of genes in *P. malariae* whose gene ID is annotated as either *pir, stp1, tryptophan-rich, etramp, phist, fam-l, or fam-m*. These do not include pseudogene annotations. Corresponding values were determined for *P. brasilianum* through matching annotations obtained by Orthofinder.

The 14 mapped chromosomes and the organellar genomes were further evaluated using the LastZ aligner^16^ in the Geneious suite^17^. Out of the 29.1 Mb reference *P. brasilianum* genome, 88.4% was aligned between both genomes by the LastZ alignment and 98.4% of these nucleotides were identical (Table S1). Alignments done with Mauve^18^ demonstrated that most genomic differences between the two genomes were localized in the telomeric ends of chromosomes (data not shown).

**Figure 2:**
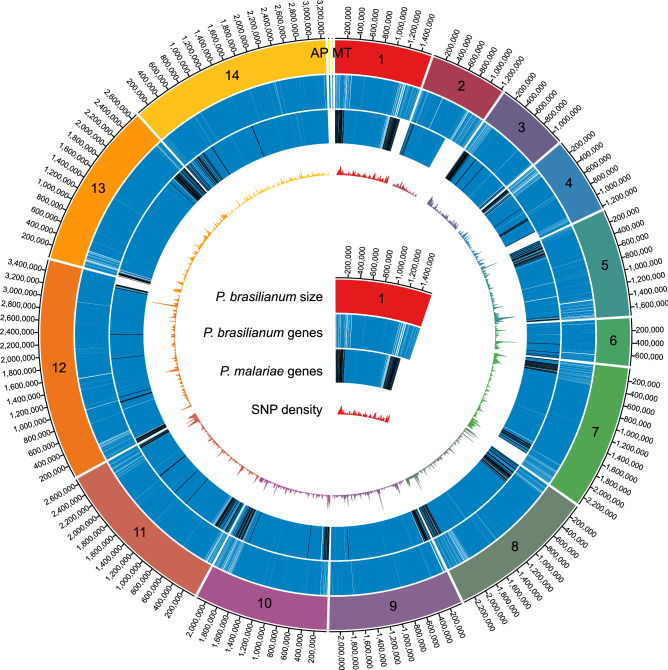
Genome organization of *P. brasilianum* and its variation from *P. malariae.* The four concentric rings, from outermost to innermost, show (1) the sizes and labels of the 14 *P. brasilianum* chromosomes, as well as the Apicoplast and Mitochondria; (2) the location of the 5,559 *P. brasilianum* genes (blue), excluding those on unplaced scaffolds; (3) the location of the 5,010 *P. malariae* genes (blue) and pseudogenes annotated onto the *P. brasilianum* genome through 80% or higher sequence similarity, excluding those on unplaced scaffolds; (4) line plot indicating SNP density of the 41,125 sequence variants between *P. brasilianum* and *P. malariae* sequences aligned by LASTZ alignment. The chromosome sizes and SNP density tracks are labeled with corresponding colors. The figure was generated using the Circa software.

As described in the methods, we used GeneMarkES^19^ and Augustus^20^, along with manual curation, to annotate 5924 genes, 144 of which belonged to the *pir* gene family, 213 to *fam-l* gene family, and 157 to the *fam-m* gene family. While structurally, we find that *P. malariae* and *P. brasilianum* share a high degree of similarity, we observe subtle differences when comparing the two species' overall gene content and identity (Fig. 2). A total of 5559 (93.8%) *P. brasilianum* genes were annotated across the 14 *P. brasilianum* chromosomes. By comparison, in *P. malariae* a total of 5,458 (83.0%) genes were annotated across the 14 *P. malariae* chromosomes. Throughout both genomes, a total of 4,970 (75.5%) *P. malariae* genes and pseudogenes had 80% or higher sequence similarity with 4,864 (82.1%) *P. brasilianum* genes.

### Evolutionary characteristics of *Plasmodium brasilianum*

**Figure 3:**
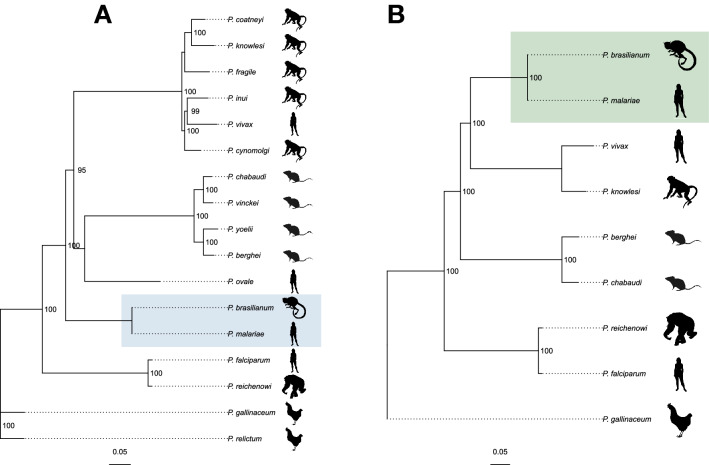
Phylogenetic distances among *Plasmodium* species visualized by orthologous genes. Multiple sequence alignment was performed across *Plasmodium* species and an evolutionary tree was generated using the PROTGRAMMAAUTO model of RaxML. (**A**) Phylogenetic tree from a sequence alignment using 82 orthologous groups (1,567 genes) found in all 19 *Plasmodium* species with an available draft/complete genome. (**B**) Phylogenetic tree from a sequence alignment using 90 orthologous groups (815 genes) found in nine representative *Plasmodium* species^7^. To the right of each phylogenetic tree is a visual depiction of the primary vector each *Plasmodium* species infects during the parasite’s asexual life cycle. Colored boxes (blue in A and green in B) depict the locations of *Plasmodium brasilianum* and *Plasmodium malariae*.

To further understand the divergence between *P. brasilianum* and *P. malariae*, we identified 92 orthologous groups that have a crucial role in the invasion of erythrocytes^21^ (Table S2). There were 82 orthologous groups (1,567 genes) that have an ortholog in all currently available draft or complete *Plasmodium* genomes. An evolutionary tree was constructed with protein sequences from these genes using RaxML^22^ to better understand the evolutionary relationship of *P. brasilianum* with other *Plasmodium* species (Fig. 3A). The tree shows that *P. malariae* is the closest related species to *P. brasilianum*. The same result is seen using a subset of 90 orthologous groups (815 genes) that have an ortholog in the nine species previously considered ^7^ (Fig. 3B).

### Comparative analysis between human malaria parasite *Plasmodium malariae *and *Plasmodium brasilianum*

From the genomic characteristics and evolutionary analysis described above, we observed that *P. brasilianum* and *P. malariae* share 97% pairwise identity as determined by LastZ alignments. Despite this, most recorded *P. brasilianum* infections have been isolated to new world monkeys, while *P. malariae* is predominantly found to infect humans in South America and Asia. To evaluate if there is sequence evidence for host specificity, we compared protein sequences from all available *P. malariae* and *P. brasilianum* accessions of 20 genes previously reported to affect host and vector invasion^23^ (Table 2). Of these, three genes had insufficient accessions to evaluate sequence variations, eight genes had minor or no sequence variation, and nine genes had sequence variations among accessions of the two species. Out of those nine genes, the *csp* gene is the most studied and was further evaluated.

**Table 2:** Comparative analysis of genes related to vertebrate host and vector invasion.

Gene name	References	Function	Vector stage	Host stage	Sequence variation
*p25*	^73^	Laminin interaction	Yes	Yes	No
*P28*	^74^	Laminin interaction	Yes	Yes	No
*soap*	^75^	Secreted ookinete adhesive protein	Yes	No	Yes
*ctrp*	^76^	Ookinete invasion CTRP (circumsporozoite & TRAP-related protein)	Yes	No	Yes
*csp*	^77,78^	Sporozoite coat protein circumsporozoite protein	Yes	No	Yes
*sr*	^79^	Scavenger receptor-like protein	Yes	No	No
*imc1*	^80^	Inner membrane complex, involved in invasion into salivary glands	Yes	No	No
*ecp1/ser8*	^81^	Egress cysteine protease 1/ serine repeat antigen, involved in sporozoite egress	Yes	No	Yes
*sera4*	^82^	Serine repeat antigen, upregulated prior to merozoite formation	No	Yes	Yes
*sera5*	^82^	Serine repeat antigen, upregulated prior to merozoite formation	No	Yes	Yes
*maebl*	^83–85^	Sporozoite adhesion	Yes	Maybe	Yes
*trap*	^86–89^	Thrombospondin-related anonymous protein, mediates invasion to salivary gland and hepatocytes	Yes	Yes	Yes
*sgs1*	^90^	Salivary gland surface protein	Yes	No	Yes
*uis10*	^91^	Encodes a secreted phospholipase	No	Yes	No
*uis3*	^92^	Liver stage development and maturation	No	Yes	No
*uis4*	^93^	Liver stage development and maturation	No	Yes	No
*p36p*	^94,95^	Hepatocyte invasion and liver stage maturation	No	Yes	
*spect*	^96^	Sporozoite transmigration	No	Yes	
*spect2/plp1*	^97^	Sporozoite transmigration	No	Yes	
*msp1*	^98^	Merozoite surface protein 1	No	Yes	No

A list of 20 genes related to vertebrate host and vector invasion were obtained through literature searches. For each gene, its gene name, literature reference, function, and whether the gene product functions during the vector and/or host stage of the parasite’s life cycle are described. The final column lists whether the protein sequences from *P. malariae* and *P. brasilianum* accessions vary between the two species or not, or if there were not sufficient accessions to evaluate using multiple sequence alignment (blank).

**Figure 4:**
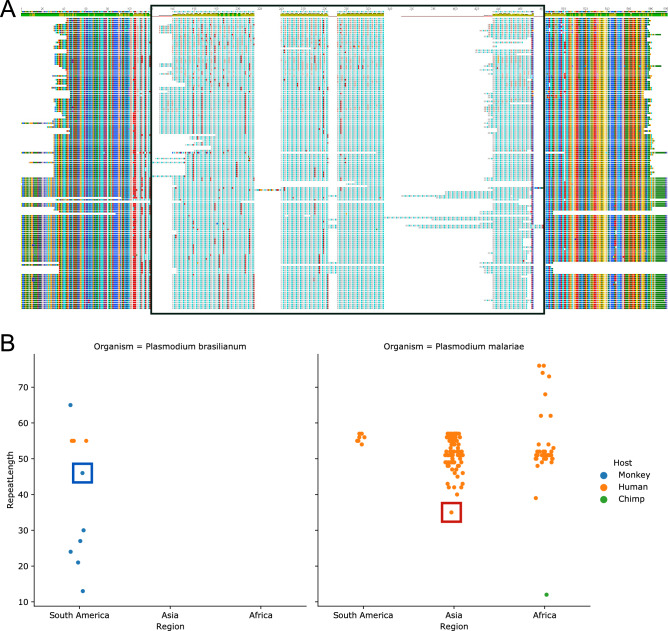
Csp tetrapeptide repeat length variation among *P. brasilianum* and *P. malariae* samples. (**A**) Multiple Sequence Analysis of the circumsporozoite protein Csp annotations from NCBI for *P. malariae* and *P. brasilianum*. The boxed-out region indicates the location of a tetrapeptide repeat (NAAG/NDAG/NAPG/NDEG) with variable length among the samples. (**B**) Comparison of *csp* repeat lengths in nucleotides between *P. malariae* and *P. brasilianum* in samples from different continents and hosts. The *P. brasilianum* sample from this study and the *P. malariae* sample from Rutledge et al.^7^ are labeled with a blue and a red box, respectively.

The CSP protein plays a crucial role in the parasite’s invasion into the salivary glands of the vector^24^. Comparing our csp sequence along with all the published sequences for *csp* from *P. brasilianum* and *P. malariae* (Table S3), we observed variability in the large tetrapeptide repeat (NAAG/NDAG/NAPG/NDEG) contained within the gene, whereas the non-repeat regions of the gene were almost identical (Fig. 4A). Comparing the lengths between the two species, we see that the tetrapeptide repeat length in *P. malariae* from infected humans from Asia and South America lies in the range of 55–60 nucleotides, while the sequences from Africa show greater variability in repeat lengths (Fig. 4B). By contrast, *csp* sequences from non-human primate infected parasites, namely a Chimpanzee infected *P. malariae* and seven sequences from New World monkey infected *P. brasilianum* samples, had shorter repeat segments than human infected *P. malariae* and *P. brasilianum* samples. This presents an interesting finding, suggesting that CSP may play a role in host specificity and adaptation and provides a direction to pursue in future studies.

**Figure 5:**
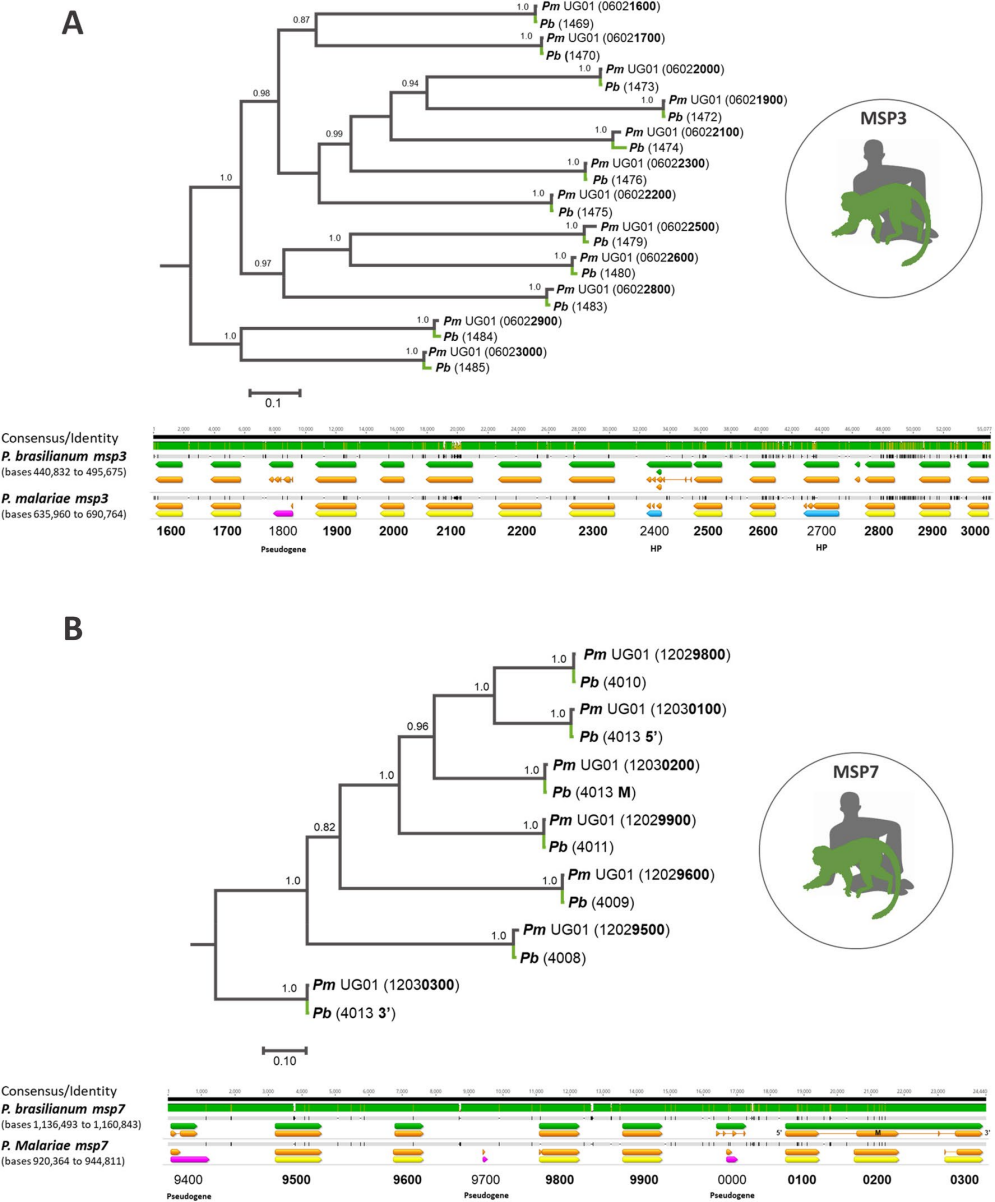
Phylogenetic distances and genome organization of *msp3* and *msp7* regions. Multiple sequence alignment was performed using members of the merozoite surface protein (*msp*) genes and a phylogenetic tree was generated using the Bayesian method implemented in MrBayes v3.2.6. (**A**) Phylogenetic tree (top) from a sequence alignment using 12 *P. malariae msp3* genes and their corresponding sequence matches in *P. brasilianum*. Branches from *P. malariae* genes are grey with their unique ID label shown in bold, and branches from *P. brasilianum* are green. Genome browsers snapshot (bottom) of the *msp3* region from both species. Genes demonstrated in the phylogenetic tree are in bold. Consensus between the two genomes is shown at the top of the browser shot with differences depicted as dips in the green heatmap and as black marks in the grey tracks for each species. (**B**) Phylogenetic tree (top) and genome browser snapshot (bottom) from a multiple sequence alignment using 6 *P. malariae msp7* genes and their corresponding sequence matches in *P. brasilianum*. (*Pm* = *Plasmodium malariae*, *Pb* = *Plasmodium brasilianum*; Green = *P. brasilianum* genes, orange = coding sequences, yellow = *P. malariae* protein coding genes, pink = *P. malariae* pseudogenes, blue (HP) = *P. malariae* hypothetical proteins; 5′ = 5′ end coding sequence, M = middle coding sequence, 3′ = 3′ end coding sequence).

In addition to *csp*, we evaluated the genome organization and sequence similarities between the *P. brasilianum* and *P. malariae* merozoite surface protein (MSPs) genes. MSPs genes mediate the initial contact stage of erythrocyte invasion by the parasite^25^. Of particular interest are the multifamily gene members of MSP3 and MSP7, each of which are organized into a contiguous genomic region and have been pursued as potential vaccine candidates^26,27^. MSP1 and MSP9 gene sequences from *P*. *malariae* were also compared and found to be most similar to their *P. brasilianum* ortholog, but since they make up the sole members of their respective families they were not analyzed further. Each of the 12 *msp3* genes (Fig. 5A, top) and the seven *msp7* genes (Fig. 5B, top) from *P. malariae* were most similar to their *P. brasilianum* ortholog. Furthermore, the *msp3* (Fig. 5B, bottom) and *msp7* (Fig. 5B, bottom) gene regions were organized the same way in both genomes. This tendency for *msp3* or *msp7* paralogs to be organized in a clustered array on a chromosome is not surprising as it has been previously reported for both gene families in most *Plasmodium* species with a completely annotated genome^28,29^. The notable exception is that three *P. malariae* genes (PmUG01_12030100, PmUG01_12030200, PmUG01_12030300) aligned to a single *P. brasilianum* gene (gene4010). Closer examination revealed better overlap with these genes with the three different coding sequences of gene4010, pointing to differences in annotations for nearly identical sequences between the two genomes. Further work utilizing RNA-based sequencing experiments is needed to validate and optimize these annotations.

## Discussion

Malaria remains a major public health issue affecting 85 countries with endemic disease contributing to an estimated 241 million cases and 627,000 deaths in 2020^1^. Significant gains have been made in reducing malaria burden using traditional malaria control tools, although recently progress has slowed. Introduction of novel tools, including the introduction of the RTS,S/AS01 malaria vaccine that targets the circumsporozoite protein of *P. falciparum*, may help enhance efforts to reduce malaria transmission^30^. In addition to vector control tools, case management remains an important pillar for malaria control and elimination. As progress is being made in eliminating malaria in Southeast Asia, zoonotic hosts of malaria parasites like *P. knowlesi* have become large reservoirs of malaria and are challenging malaria elimination efforts^31–33^. Simple diagnostic tools such as microscopy and rapid diagnostic tests are not sufficiently sensitive to make accurate diagnosis of *P. knowlesi*; molecular tools are necessary for diagnostic confirmation. Even with powerful protocols for rapidly isolating DNA, creating purified libraries, and obtaining millions of next generation sequenced reads, the obtained sequences are not informative if the targeted sequence is the same in multiple species, as would be the case for *P. malariae* and *P. brasilianum* targets that are nearly identical, or if the targeted sequence is not known for species that may be present in the sample. In this context, the availability of a *P. knowlesi* genome sequence allowed for the identification of better diagnostic targets^34^.

Furthermore, zoonotic malaria cases caused by zoonotic *Plasmodium* species are expected to rise, increasing the need for accurate diagnosis. This is due to two main factors: (1) human contact with nonhuman primate *Plasmodium* reservoirs, facilitated by mosquito vectors, is expected to rise in areas of the world where deforestation increases the probability of zoonotic transmission; (2) surveillance and treatments focused on eliminating malaria caused by human-infecting *Plasmodium* species may create an opportunity for infections in humans by zoonotic *Plasmodium* species^35^. For example, Malaysia has reported zero indigenous non-zoonotic malaria cases in the last four years but has struggled with cases of zoonotic malaria due to *P. knowlesi*^1^. Experimentally, several zoonotic malaria parasites were able to be transmitted to humans by mosquito bites, including *P. knowlesi* (macaque monkeys of Southeast Asia)^36,37^, *P. cynomolgi* (macaque monkeys of Asia)^38–40^, *P. brasilianum* (multiple platyrrhine monkey species of South and Central America) ^40^, and *P. inui* (several monkey species of Asia)^41^. A naturally acquired human infection has been reported for each of these species since: *P. knowlesi*^42^, *P. cynomolgi*^4,43^, *P. simium*^44^, *P. brasilianum*^5^, and *P. inui*^4,45^.

Accurate detection of parasites in these zoonotic infections in humans is important for surveillance and relies on conserved primers, able to bind to genomes of multiple species, to amplify genes or genomic stretches of interest from extracted DNA for use in sequencing or restriction digestion-based identification assays. Deciding which targets to utilize for accurate identification can be greatly improved by the availability of complete reference genomes for these species. Reference genomes exist for *P. knowlesi* (strain A1-H.1^46^, strain H^47^, and strain Malayan Strain Pk1 A^48^), *P. cynomolgi* (strain B^49^ and strain M^50^), *P. inui* (strain San Antonio 1, GCA_000524495.1), and *P. simium*^51^. The first complete genome for *P. brasilianum* reported here provides the toolkit needed for accurate detection and understanding of zoonotic malaria from these emerging sylvatic reservoirs^52^.

Rutledge and colleagues published a complete genome in 2017 for *P. malariae* whose 18S rRNA sequences were indistinguishable from *P. brasilianum*^7^. The similarity in the 18S gene sequences between the two species has been observed before. In addition, the similarity in *csp* sequences between the two species further emphasized the lack of genomic distinction^53^. Nonetheless, *P. brasilianum* and *P. malariae* have been maintained as distinct species since 1931. This was based on an experiment by Clark and Dunn, who demonstrated that seven patients given parasitized blood from a black spider monkey did not show evidence of successful infection^54^. The recent finding of a natural infection of *P. brasilianum* in humans disputes the notion that *P. malariae* and *P. brasilianum* are simply closely related and separated based on host specificity^5^. In fact, genomic comparisons support the hypothesis that *P. brasilianum* and *P. malariae* may be the same species^5^. Indeed, *P. malariae* has been found infecting African apes demonstrating the plasticity of this species in terms of the hosts it can infect^55^.

We observed 97.3% pairwise identity from LastZ alignments between our chromosomes and organellar genomes and those assembled for *P. malariae* by Rutledge et al.^7^ (Table S1). From these pairwise analyses, we identified a total of 41,125 non-synonymous SNPs between *P. malariae* and *P. brasilianum* (Table S4). These nucleotides constitute less than one percent of the genome. In fact, a total of 98.4% of the aligned regions between the two genomes were identical (Table S1). Many of the observed differences can be attributed to the disparity in the number of non-chromosomal contigs and the annotations for each genome. The *P. brasilianum* genome has 29 unplaced contigs, compared to 47 from *P. malariae*. Overall, the *P. brasilianum* genome has a higher resolution, with more telomeric sequences mapped. The telomere ends represent a challenging chromosomal region for assembly, and we utilized optical maps during contig assembly to assist with assembly at these regions^56^. We observed a total of 4,970 *P. malariae* genes that had 80% or higher sequence similarity with 4864 *P. brasilianum* genes (Table S5). Several *P. brasilianum* genes overlapped with more than one *P. malariae* gene. This can be seen in our *msp7* comparisons (Fig. 5B), where a single *P. brasilianum* gene overlaps with three *P. malariae* genes. We cannot distinguish which annotation is more accurate without real expression data.

Gene annotations could be improved by incorporating RNA-seq expression data from *P. brasilianum* to annotate its transcriptome. Depending on the experimental design, a transcriptome typically only represents a snapshot of expression specific to a given cell type, developmental stage, or external stimulus. However, this can be addressed by utilizing single-cell RNA sequencing (scRNA-seq), as^58^. In addition to providing readouts for genes expressed only during specific stages of development, incorporating scRNA-seq would expand upon alternative transcript annotations. Otherwise, High-throughput Chromosome Conformation Capture (Hi-C) technologies could be utilized, alongside additional optical mapping experiments, to resolve the mapping of unplaced scaffolds^48^.

In conclusion, this work provides the first complete reference genome of *P. brasilianum*. While our findings support the hypothesis that *P. malariae* and *P. brasilianum* are strains of the same species, their evolutionary relationship cannot be fully determined without additional genome sequences from additional isolates. The notable exception to this was our observation that *csp* repeat sequences from non-human infecting *P. malariae* and *P. brasilianum* samples were shorter compared to those from human infections (Fig. 4B). Further studies are needed to evaluate the significance of this finding.

## Methods

### Sample origin and DNA isolation

Genomic DNA was extracted from ex vivo mature schizont stage parasites of the Bolivian I strain of *P. brasilianum* using the Qiagen DNA Blood Kit (QIAGEN, CA, USA, cat:51,104) and the MagAttract high-molecular-weight (HMW) DNA kit (Qiagen, MD, USA, cat: 67,563).

### Sequencing

Genomic DNA libraries were prepared from isolated DNA using the NEBNext Ultra library prep kit (New England Biolabs, MA, USA, cat: E7370S) and the standard PacBio 20-kb procedure (Pacific Biosciences, CA, USA). Two separate runs were performed on the MiSeq using the MiSeq 500 cycle reagent kit (Illumina, CA, USA, cat:MS-102–2003). Two additional runs were performed on the PacBio RSII with C4 v2 chemistry for 360-min movies (Pacific Biosciences, CA, USA).

### Quality filtering

FastQC^57^ was performed on the sequenced reads to analyze the quality of the Illumina and the Pacbio sequences. Raw reads were aligned against the host genome (GenBank accession no. 1GCA_000235385.1) and the reads that mapped to the host genome were discarded. Adapter trimming and quality control of the Illumina sequences were performed on the remaining reads using BBDuk from the BBMap toolkit^58^. Sequences with mean quality lower than 30, and length less than 50 were discarded from further analysis.

### Genome assembly

The MaSuRCA hybrid genome assembler^12^, with default settings, was used to assemble the two Pacbio sequenced libraries and the Illumina paired-end sequenced libraries. Overlapping contigs from the MaSuRCA assembly were merged using Circlator^13^. Since the *P. brasilianum* genome and the mitogenome are linear genomes, with the apicoplast organized into a circular genome, Circlator was used to merge overlapping contigs into extended linear contigs^59,60^. The IPA script (https://github.com/ThomasDOtto/IPA) was run on the extended contigs to filter out contigs smaller than 5 kb. Contigs that are contained up to 90% within another contig were removed. Contigs with Illumina coverage greater than 50% of median coverage were merged. The resulting contigs were ordered and oriented with Abacas2 (https://github.com/satta/ABACAS2) by using the *P. malariae* genome as a reference. Finally, PILON^15^ was used for SNP calling and error-correcting in contigs. The apicoplast and mitochondrial genomes from the prior draft assembly^11^ were added to the extended, corrected assembly to generate the final complete assembly.

### Genome annotation

Gene annotation was performed using both the de novo gene prediction tool GeneMark ES^19^, and Augustus^20^ that was trained on gene models from *P. vivax*. The resulting gene models were mapped against NCBI’s RefSeq Non-redundant protein database using the Diamond protein aligner^61^, in addition to manual curation. Orthologs across all 19 known *Plasmodium* species were identified using Orthofinder^62^. Blast2Go^63^ was used to perform GO enrichment analysis and Pfam annotations.

### Annotation comparison

The Geneious Prime suite (Geneious Prime 2021.2.2. (https://www.geneious.com)) was used to visually compare the annotations between the *P. brasilianum* genome and the *P. malariae* genome from Rutledge et al.^7^. Each *P. malariae* chromosome that was structurally complete from end to end was aligned to its corresponding *P. brasilianum* chromosome one at a time using LastZ^16,64^, with default parameters and by using the *P. brasilianum* chromosome as the target sequence (Table S1). SNPs (Table S4) were identified from these alignments using Geneious’s “Find Variations/SNPs” option, with the options set to only find variants Inside CDS with a minimum coverage of 1. Synonymous mutations were excluded from the results. To visually place where each *P. malariae* gene annotation was aligned to the corresponding chromosome in *P. brasilianum*, the “Live Annotation and Predict” option in Geneious was used to transfer annotations from *P. malariae* chromosomes onto corresponding *P. brasilianum* chromosomes when the annotation’s sequence had at least 80% similarity. Overlapping genes were organized into a translation sheet for quick 1:1 comparison between the two genomes at these highly similar sequence regions (Table S5). A circa plot (Fig. 2) was created to demonstrate the size of each non-scaffold *P. brasilianum* chromosome, *P. brasilianum* gene annotations, transferred annotations from *P. malariae*, and SNP locations using the Circa software (Circa, available at http://omgenomics.com/circa).

### Comparative genomics

We identified 92 orthologous groups that have at least one ortholog in most of the *Plasmodium* genomes that have a crucial role in the invasion of erythrocytes^21^ (Table S2). For all 19 currently available draft or complete genomes, there were 82 orthologous groups (1567 genes) that have an ortholog in all 19 genomes. When the genomes are restricted to only those species that were considered by Rutledge et al.^7^, namely *P. vivax*, *P. brasilianum*, *P. berghei*, *P. falciparum*, *P. knowlesi*, *P. reichenowi*, *P. chabaudi*, *P. gallinaceum*, and *P. malariae*, a total of 90 orthologous groups (815 genes) were found across in all nine genomes. Multiple sequence alignment using these 82 or 90 orthologous groups was performed using MUSCLE across all 19 or 9 *Plasmodium* species using Gblocks^65^. RaxML^22^ was used to generate evolutionary trees using the PROTGAMMAAUTO model to learn the likelihood model for the alignments.

To understand the difference in host interaction between the closely related species *P. brasilianum* and *P. malariae*, gene sequences from previously reported genes^23^ annotated as functionally important for host and vector invasion were compared among accession from *P. brasilianum* and *P. malariae*. The circumsporozoite adhesion protein coded for by the *csp* gene had multiple accessions for analyses for both species and had ample protein sequence variation among the accession and was studied further (Table S3). Multiple sequence alignment was performed using MUSCLE v3.8.425^66^, and the difference in the characteristic sequence repeats in the CSP protein was visualized with the Geneious Alignment View tab after using the Geneious Tree Builder with default parameters, as well as the Seaborn^67^ catplot function.

Additionally, several members of the merozoite surface protein gene families were compared, most notably *msp3* and *msp7*. Gene sequences from *P. malariae* were aligned to corresponding gene chromosomes in *P. brasilianum* with the Geneious read mapper to identify the corresponding orthologs. The bedtools^68^ “getfasta” function was used to extract the sequence for each gene from both species (Table S6), and multiple sequence alignment was performed using ClustalX v2.0.12^69^ and MUSCLE as implemented in SeaView v4.3.5^70^. Phylogenetic trees were generated using the Bayesian method implemented in MrBayes v3.2.6 with default parameters^71^, including a general time-reversible model with gamma-distributed substitution rates and a proportion of invariant sites (GTR + Γ + I). This was the best model that fit the data with the lowest Bayesian Information Criterion (BIC) scores, as estimated by MEGA v7.0.14^72^. Bayesian support was inferred for the nodes in MrBayes by sampling every 1,000 generations from two independent chains lasting 2 × 10^6^ Markov Chain Monte Carlo (MCMC) steps. The chains were assumed to have converged once the potential scale reduction factor (PSRF) value was between 1.00 and 1.02, and the average SD of the posterior probability was < 0.01. Once convergence was reached as a “burn-in,” 25% of the samples were discarded.

## Supplementary Information

Supplementary Information 1.

Supplementary Information 2.

Supplementary Information 3.

Supplementary Information 4.

Supplementary Information 5.

Supplementary Information 6.

Supplementary Information 7.

## Data Availability

The assembled genome sequences generated during the current study are available in the NCBI repository (Bioproject PRJNA810024, BioSample SAMN26224112, Assembly GCA_023973825.1).
