# Supplementary material for: The first complete genome of the simian malaria parasite Plasmodium brasilianum
Source: Sci Rep. 2022 Nov 17;12:19802. doi: 10.1038/s41598-022-20706-6 (PMC9671904; doi:10.1038/s41598-022-20706-6)
Supplement: Supplementary file 1 — Supplementary Information 1. [file 41598_2022_20706_MOESM1_ESM.pdf]

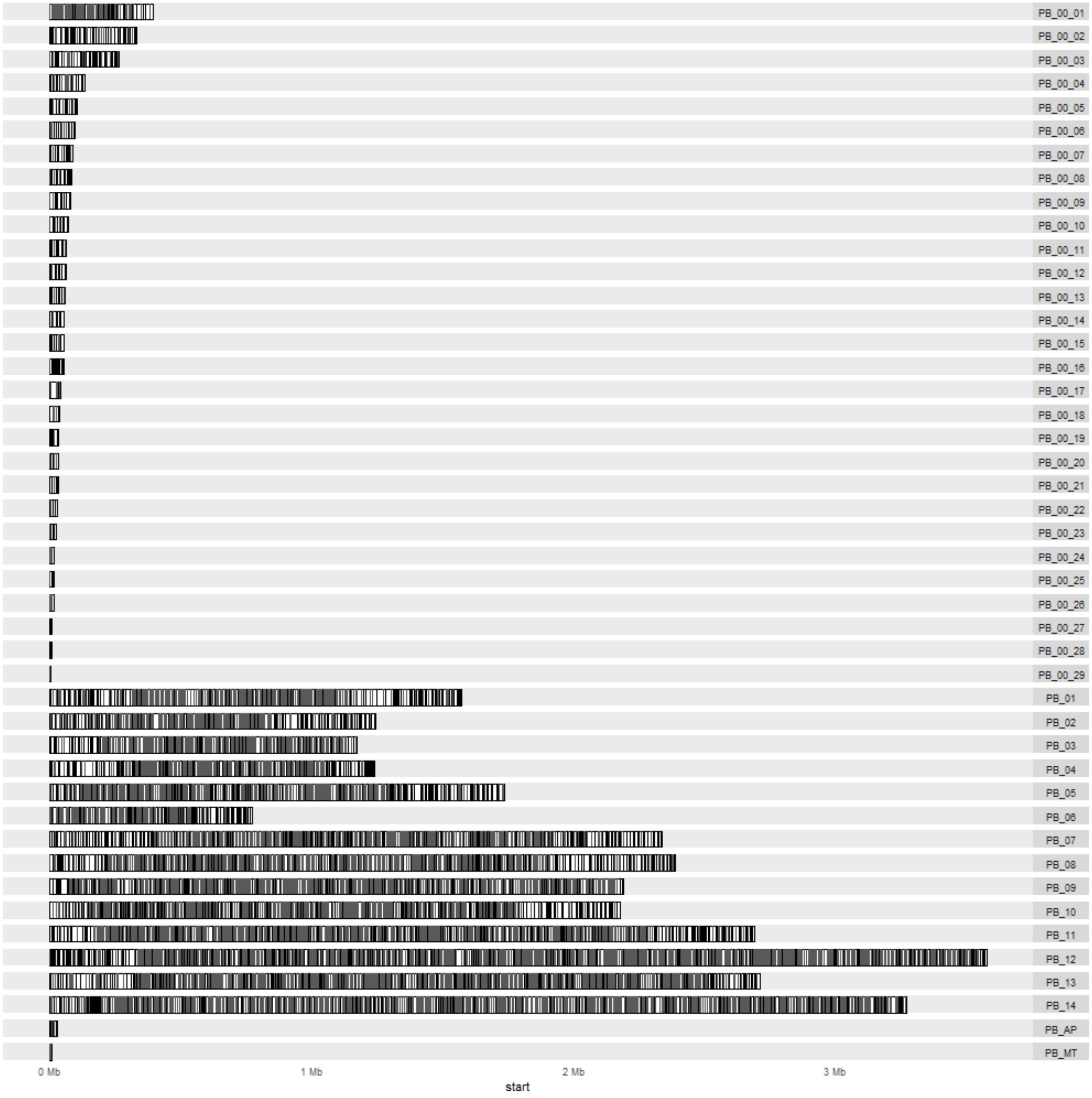

Figure S1. Karyogram of the final set of contigs utilized for gene annotation. Contigs denoted with "_00_" constitute scaffolds that could not be reliably placed on any nuclear, apicoplast, or mitochondrial genome.
